# Homoeologous Exchanges, Segmental Allopolyploidy, and Polyploid Genome Evolution

**DOI:** 10.3389/fgene.2020.01014

**Published:** 2020-08-28

**Authors:** Annaliese S. Mason, Jonathan F. Wendel

**Affiliations:** ^1^Plant Breeding Department, Justus Liebig University Giessen, Giessen, Germany; ^2^Ecology, Evolution, and Organismal Biology Department, Iowa State University, Ames, IA, United States

**Keywords:** polyploidy, homoeologous exchanges, chromosome behavior, synthetics, genome evolution

## Abstract

Polyploidy is a major force in plant evolution and speciation. In newly formed allopolyploids, pairing between related chromosomes from different subgenomes (homoeologous chromosomes) during meiosis is common. The initial stages of allopolyploid formation are characterized by a spectrum of saltational genomic and regulatory alterations that are responsible for evolutionary novelty. Here we highlight the possible effects and roles of recombination between homoeologous chromosomes during the early stages of allopolyploid stabilization. Homoeologous exchanges (HEs) have been reported in young allopolyploids from across the angiosperms. Although all lineages undergo karyotype change via chromosome rearrangements over time, the early generations after allopolyploid formation are predicted to show an accelerated rate of genomic change. HEs can also cause changes in allele dosage, genome-wide methylation patterns, and downstream phenotypes, and can hence be responsible for speciation and genome stabilization events. Additionally, we propose that fixation of duplication – deletion events resulting from HEs could lead to the production of genomes which appear to be a mix of autopolyploid and allopolyploid segments, sometimes termed “segmental allopolyploids.” We discuss the implications of these findings for our understanding of the relationship between genome instability in novel polyploids and genome evolution.

## Introduction

Recent technological advances have vastly expanded access to genomic information, even for complex genomes (reviewed by Yuan et al., 2017). As an adjunct to *de novo* genome assembly for the creation of reference genomes, population genomic studies enable resequencing of multiple individuals within species to provide genetic data on a scale and at a resolution only dreamed of just a few years ago. In this new genomics era, it seems timely to revisit some of the fundamental concepts established in the early years of cytogenetics, particularly regarding insights into meiosis in polyploids and how this new understanding helps predict and explain several aspects of polyploid evolution and diversification.

In this review, we provide an overview of the cytogenetic processes associated with polyploidy, particularly the early stages of polyploid formation, and how these processes may induce genomic structural variation and give rise to novel phenotypes, thus providing an evolutionary substrate for diversification. We revisit the idea that homoeologous recombination in polyploids may lead to rapid karyotypic and genomic restructuring in the first few generations after polyploid formation (Song et al., 1995), and in the process generate duplicated genomic regions and other findings that may seem difficult to explain based on species relationships and phylogenetic inferences. We further discuss how homoeologous exchanges can affect phenotypes to further impact the process of speciation via saltational changes. Lastly, we introduce the concept that homoeologous exchanges may be responsible for the observation of “segmental allopolyploidy,” where auto- and allopolyploidy appear to both be present across the polyploid genome (Stebbins, 1950; Sybenga, 1996).

## The Polyploid Spectrum: From Auto to Allo

Polyploidy, where three or more haploid chromosome sets are present within a single organism, is ubiquitous across the plant and animal kingdoms, with the minor exception of mammal and bird lineages (Van De Peer et al., 2017). Polyploids were first classified into “autopolyploids” and “allopolyploids” nearly a century ago by Kihara and Ono (1926), who proposed the distinction that autopolyploids derive from chromosome doubling of a single individual, and allopolyploids derive from hybridization. However, although chromosome doubling within reproductive tissue of a single individual may yield two identical chromosome complements, the frequency of this route to polyploidy remains unclear. As early as 1947, Stebbins cast doubt on the existence of natural autopolyploids formed via chromosome doubling (Stebbins, 1947). We now know that newly formed polyploids that arise via chromosome doubling are likely to suffer inbreeding depression (Abel and Becker, 2007), with major advantages conferred by heterozygosity in both auto- and allopolyploid species (for review see Bingham, 1980). Hence, it seems likely that most autopolyploidy events actually occur via sexual reproduction between two individuals within a species (for review see Soltis et al., 2014; Spoelhof et al., 2017b), or at the very least via meiotic events which allow for the generation of novel variation in progeny (for review see De Storme and Mason, 2014). In fact, the mechanism of “hybridization followed by chromosome doubling” was re-evaluated 45 years ago by Harlan and DeWet (1975), who pointed out that the vast majority of hybridization events rely on meiotic, rather than mitotic, mechanisms, i.e., unreduced gametes rather than mitotic errors. This viewpoint has been reinforced in the intervening years (Ramsey and Schemske, 1998; De Storme and Geelen, 2013; De Storme and Mason, 2014; Mason and Pires, 2015).

Irrespective of the mode of formation, the terms “allopolyploidy” and “autopolyploidy” clearly represent two ends of a cytogenetic and taxonomic conceptual continuum with broadly overlapping suites of characteristic features (Wendel and Doyle, 2005; Carputo et al., 2006). In recent years, the taxonomic definition for autopolyploidy, as arising within a species, and allopolyploidy, as forming between species, has predominated (Spoelhof et al., 2017b). This may be the most useful definition, despite species concept and classification difficulties, particularly for autopolyploid species (Soltis et al., 2007; Barker et al., 2016). Allopolyploidy events can also vary greatly in the amount of divergence between the progenitor genomes. For example, some interspecific hybridization events that lead to “allopolyploidy” may involve species with subgenomes that are less diverged from each other than “autopolyploid” events arising within a highly polymorphic single species. In rice (*Oryza sativa*), for example, hybridization between the two subspecies *japonica* and *indica* to form novel polyploids results in “genomic shock” and allopolyploid-style gene expression partitioning (Zhao et al., 2018), a phenomenon more normally attributed to allopolyploidy (Grover et al., 2012). By contrast, hybridization between taxonomic species in the *Brassica* “C genome” cytodeme can often lead to fully or partially fertile hybrids with predominantly homologous chromosome pairing during meiosis (Kianian and Quiros, 1992; Bothmer et al., 1995).

In addition to auto- and allo-, Stebbins (1947) proposed a new category of polyploids, known as “segmental” allopolyploids. Stebbins actually used both chromosome behavior and genome structural divergence concepts in his application of the term, as at the time chromosome pairing was thought to rely solely on “structure,” rather than sequence homology. He first mentions that “*Cytologically, [segmental allopolyploids] are characterized by the presence of multivalents in varying numbers, so that in meiosis they often resemble autopolyploids more than true allopolyploids*.” He later states that “*A segmental allopolyploid may, therefore, be defined as an allopolyploid of which the component genomes bear the majority of their chromosomal segments in common, so that the diploid hybrid from which it is derived has good pairing at meiosis, but in which these genomes differ from each other by a large enough number of chromosomal segments or gene combinations so that free interchange between them is barred by partial or complete sterility on the diploid level*.” This latter idea, that of intermediacy between the archetypal poles of autopolyploidy and allopolyploidy, has often been an unstated assumption in the application of the term since Stebbins’ first use, rather than the operational definition that segmental allopolyploids show both multivalent and bivalent formation for some portions of the chromosome complement. Today, however, we would most likely characterize these cases as autopolyploids. As pointed out by Sybenga a quarter century ago (Sybenga, 1996), newly formed polyploids often display multivalents, but established autopolyploids instead are characterized by bivalent formation with random partner choice (tetrasomic inheritance).

This distinction between newly formed vs. evolved is important, as it illustrates the connectedness of the terms auto- and allopolyploidy as well as a temporal dimension. That is, autopolyploids may form between divergent germplasm groups within a species, but later evolve fully disomic inheritance and become “allopolyploid-like” (Bingham, 1980), and not necessarily at homogeneous rates throughout the genome. Thus, it seems important to distinguish mode of formation and evolved meiotic behavior.

## Mechanisms of Genome Stabilization in Polyploids

Newly formed polyploids, whether autopolyploids or allopolyploids, face a major challenge in becoming established, that of regulating meiosis (reviewed by Pelé et al., 2018). Meiosis is a tightly controlled process in all organisms, as fertile progeny must be formed from recombinationally variable gametes. This tight regulation commonly breaks down when two genomes are suddenly present instead of one, i.e., when four copies of homologous chromosomes are present instead of two (reviewed by Cifuentes et al., 2010). Cytologically speaking, there are two strategies by which newly formed polyploids might regulate meiosis: “autopolyploid”-type and “allopolyploid”-type. In “autopolyploid”-type meiotic regulation, crossover number and distribution is stringently regulated: only enough crossovers are permitted so that every two chromosomes will be bound by a single crossover. This promotes strict bivalent formation (with random partner choice, i.e., tetrasomic inheritance) despite the presence of four homologous chromosomes, and is thought to be the most common method for autopolyploid meiotic regulation (Cifuentes et al., 2010; Spoelhof et al., 2017b). This is also the stability mechanism known to occur in *Arabidopsis arenosa* autotetraploids (Lloyd and Bomblies, 2016). Tetravalent formation, that is, when crossovers form between four homologous chromosomes to produce a multivalent, is rarely observed in meiosis of stable autopolyploids, and sometimes not even in synthetic autopolyploids (Sybenga, 1996).

In allopolyploid meiosis, strictly homologous pairing requires some mechanism or mechanisms to discriminate subgenomes (Stebbins, 1950). It has long been noted that newly synthesized allopolyploids suffer a higher degree of irregular chromosomal configurations than do their natural analogs, for example, in cotton (reviewed in Endrizzi et al., 1985), showing that evolutionary enforcement of homologous pairing has been selected over time. Mechanisms leading to this stabilization of homologous pairing are mostly unknown and almost certainly vary among the tens of thousands of allopolyploids that exist in plants (e.g., even B chromosomes have been implicated; Taylor and Evans, 1976). Early insights are beginning to emerge into the spectrum of possible molecular determinants of enforcement of homologous pairing. Prevention of non-homologous pairing between subgenomes in allohexaploid bread wheat is facilitated by the major qualitative effect *Ph1* locus (Sears, 1976; Feldman, 1993; Griffiths et al., 2006; Bhullar et al., 2014), for which the molecular mechanism is still not completely characterized, but which may involve suppression of *CDK2*-like activity to result in chromatin modifications (Greer et al., 2012) as well as the presence of an additional copy of a meiotic *ZIP4* gene (Rey et al., 2017). By contrast, at least eight meiosis genes have been implicated in genomic stabilization of autotetraploid *Arabidopsis arenosa* (Yant et al., 2013), with two of these genes (*ASY1* and *ASY3*) later found to directly reduce multivalent formation and chiasma number, as expected (Morgan et al., 2020). *Triticum* and *Aradidopsis* represent the two best-characterized models for meiotic regulation in polyploids to date (reviewed by Cifuentes et al., 2010; Lloyd and Bomblies, 2016). In recent years, great progress has been made toward understanding the molecular mechanisms underlying regulation of meiosis in polyploids (see reviews by Cifuentes et al., 2010; Grandont et al., 2013; Bomblies et al., 2015, 2016; Lloyd and Bomblies, 2016; Pelé et al., 2018). To date, however, only a few polyploid species and synthetics have been investigated for meiotic stability mechanisms; future investigations across the tree of life are necessary to understand the spectrum of meiotic evolutionary responses to polyploidy and which components might be generalizable.

One question of importance is whether meiotic stabilization following polyploidization is a gradual process, or whether allelic variants present in the diploid progenitors can lead to immediately stable allo- or autopolyploids. In synthetic *Brassica* hybrids, the first generation has clearly been established to be the least stable (Szadkowski et al., 2010), following which meiosis may stabilize over time (Prakash et al., 1999; Gaebelein et al., 2019), putatively due to selection for particular allelic complements conferring higher fertility (Gaebelein and Mason, 2018; Gaebelein et al., 2019). Swaminathan and Sulbha (1959) also found that stability increased over 19 generations of selection in a single genotype of autotetraploid *B. rapa* – a surprising result, because under strict self-pollination the only way for stability to arise would be via *de novo* mutation, chromosome rearrangements or changes in epimethylation, as initial plants would be 100% homozygous. However, it is clear that “complete” stabilization (i.e., complete prevention of homoeologous chromosome pairing) does not occur in *Brassica*: inspection of established *B. napus* has revealed high frequencies of chromosome rearrangements in this young allotetraploid species (Chalhoub et al., 2014; Samans et al., 2017; Mason et al., 2018). These results are similar to those found in very recent (∼80 year old) allopolyploid species in *Tragopogon*, where extensive karyotype variation has been observed, including clear products of homoeologous recombination between the subgenomes (Chester et al., 2012). In *Arabidopsis arenosa* polyploids, a gradual process of generational selection for “adapted” meiosis gene alleles has been proposed, based on selective sweeps between diploid and tetraploid populations (Yant et al., 2013). Interestingly, natural populations of tetraploid *Arabidopsis lyrata* seem to have acquired these *Arabidopsis arenosa* alleles which facilitate meiotic stabilization via interspecific hybridization (Marburger et al., 2019), suggesting a possible shortcut to stabilization.

But are all synthetic and newly formed polyploids in fact meiotically unstable? Although this seems to be a common general trend (see Pelé et al., 2018 for review), there are also examples of immediately stable auto- and allopolyploids. For instance, it seems that kale genotypes of *Brassica oleracea* can be induced to form stable autopolyploids (Jenczewski et al., 2002), despite the fact that most autotetraploids in this species are highly unstable (Howard, 1939; Zdráhalová, 1968). Gupta et al. (2016) also found stable meiotic behavior in a single genotype of *de novo* allohexaploid *Brassica* formed by the cross between *B. carinata* and *B. rapa*, despite the fact that the majority of lines from this cross combination are highly unstable (Tian et al., 2010; Gupta et al., 2016). Some allopolyploid species, such as white clover, also seem to have clear separation between subgenomes, with no indication of instability following allopolyploidization (Griffiths et al., 2019). Possibly, considerable genetic variation is present within some lineages for the frequency and prevention of non-homologous recombination events, which may occur through different mechanisms such as increased stringency of sequence-homology required for crossover formation, timing of condensation of chromosomes belonging to different genomes, and changes in crossover frequency, targeting and distribution, which may be modulated by many different genomic features and which are only now starting to be elucidated (Zhang et al., 2020; see also Cifuentes et al., 2010 for review). Although only speculation, this would perhaps explain why some plant families (e.g., Brassicaceae) have widely varying karyotypes and chromosome numbers even between closely related species (Schranz et al., 2006), while other families (e.g., Solanaceae) have highly conserved karyotypes and chromosome numbers (Wu and Tanksley, 2010).

## Homoeologous Exchanges in Allopolyploids

**FIGURE 1:**
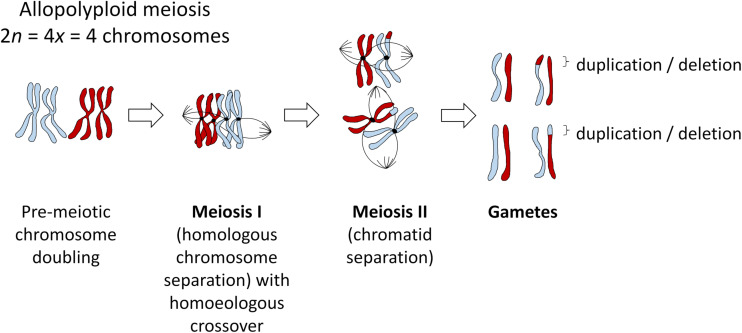
Meiosis in an example allopolyploid with 2*n* = 4*x* = 2 chromosomes (subgenomes indicated in red and blue), showing the most probable outcome of a single crossover event between homoeologous (ancestrally homologous) chromosomes. All such events will most likely be heterozygous in the first generation, even under self-pollination, as gametes with a homoeologous recombination event (duplication/deletion) will unite with gametes from a different meiosis (i.e., pollen with ovules), but may become fixed in subsequent generations after self-pollination.

Mechanisms of polyploid formation and meiotic regulation are important to consider in all polyploids, as “established” polyploid species can still be prone to meiotic errors resulting from imperfect stabilization processes. In many allopolyploid species, “homoeologous exchanges” (HEs) occur via mispairing between ancestrally related chromosomes belonging to different genomes. These exchanges swap pieces of DNA between the subgenomes, and can lead to deletions, duplications and translocations (Figure 1). Not all non-homologous exchanges are homoeologous, that is, occur between related genomic segments that have diverged from a common ancestor. Even small regions of duplicated DNA within a genome are sufficient to induce occasional non-homologous chromosome rearrangements. However, these tend to be heavily suppressed, such that recombination between repetitive sequences (which can easily result in genomic instability) is rare (Putnam et al., 2009). Hence, the vast majority of crossovers between non-homologous chromosomes occur between homoeologous regions, particularly in recent allopolyploids (Nicolas et al., 2012).

Homoeologous exchanges can result in either “balanced” or “reciprocal” translocations (“homoeologous reciprocal translocations” or HRTs) which swap the locations of two homoeologous DNA segments, or “unbalanced” or “homoeologous non-reciprocal” translocations (HNRTs) (effectively duplication/deletion events, Figure 1). However, this terminology is misleading and should probably be avoided. “Non-reciprocal” exchanges are of course actually reciprocal in terms of crossover events (see Figure 1), such that “non-reciprocal” refers only to the products of the exchange, i.e., whether one piece of DNA has been swapped for another, or whether an additional copy of a DNA sequence has replaced the homoeologous copy in a “duplication-deletion” event. Homoeologous exchanges generally occur via co-opting of the homologous recombination pathway, but where homoeologous chromosome regions (ancestrally related stretches of DNA from different subgenomes) act as the substrate instead of homologous chromosomes (Nicolas et al., 2009). Depending on the structural divergence between the subgenomes, whole chromosomes may be “homoeologous” or syntenic in terms of DNA sequence along their entire length, or single chromosomes may contain many small stretches of DNA that are homoeologous to parts of chromosomes in the other subgenome. In many species, recurrent polyploidy events have resulted in both “primary” and “secondary” homoeology: that is, homoeology between subgenomes resulting from a recent allopolyploid event, and homoeology within each subgenome between regions resulting from more ancient polyploidy events. A good example of this is provided by the *Brassica* genus: in addition to the three recent allopolyploid species *B. juncea*, *B. napus*, and *B. carinata*, with AABB, AACC, and BBCC genome complements, respectively, for which the A-B, A-C, and B-C genomic relationships, respectively represent primary homoeology, each of the A, B, and C genomes contains triplicated genomic segments resulting from mesopolyploidy events, representing secondary homoeology (Parkin et al., 2003).

Homoeologous exchanges are now known to be common in synthetic polyploids, as well as those of recent evolutionary origin. Recent allopolyploids with commonly detected HEs include *Tragopogon* species (Chester et al., 2012), peanuts (*Arachis hypogaea*; Bertioli et al., 2019; Zhuang et al., 2019), quinoa (*Chenopodium quinoa*; Jarvis et al., 2017), tobacco (*Nicotiana tabacum*; Chen et al., 2018) and rapeseed (*Brassica napus*; Chalhoub et al., 2014). Synthetics with frequent HEs include allopolyploid rice (constructed from *Oryza sativa* subsp. *indica* × subsp. *japonica*; Sun et al., 2017; Li et al., 2019) and *Brassica* species (Song et al., 1995; Gaeta et al., 2007; Szadkowski et al., 2010), as well as many more: this phenomenon may be generalizable across most newly formed polyploids as a result of meiotic instability (reviewed by Pelé et al., 2018).

It should be noted for completeness that homoeologous exchanges are not the only form of genomic instability in novel polyploids. Previously, a great deal of attention has been paid to the activation of transposable elements as a result of “genome shock,” a phenomenon first proposed by McClintock (1984). Polyploidy in many species seems to be associated with bursts of transposable element activation (for review see Vicient and Casacuberta, 2017; Nieto-Feliner et al., 2020, this issue). Transposable elements may also (rarely) act as a substrate for non-homologous recombination events (Xiao and Peterson, 2000; Xuan et al., 2012), and also cause sequence mutagenesis after excision due to double strand break repair mechanisms, which often insert or delete a few basepairs during the non-homologous end-joining process (for review see Gorbunova and Levy, 1999). Transposable element activation and novel SSR mutations have been reported in *Brassica* synthetics (Zou et al., 2011; Gao et al., 2014), and widespread loss of non-coding sequences in synthetic wheat polyploids (Ozkan et al., 2001; Shaked et al., 2001), all independent of homoeologous exchange events.

## Homoeologous Recombination Events Can Generate Novel Variation, Affect Phenotype and Act As Targets for Natural Selection

Non-homologous recombination events can result in duplications, deletions and chromosome rearrangements. Although karyotypic variation resulting from non-homologous chromosome recombination putatively occurs in almost all evolutionary lineages, facilitating karyotype change over time, it is much more likely that a chromosome rearrangement (particularly a larger deletion or duplication) will prove fatal in a diploid lineage (Schuermann et al., 2005). However, the presence of an extra set of chromosomes provides a “buffer” for chromosome change: when two or more copies of a gene or genomic region are present, this can allow novel variation to arise without impacting viability and fertility to as great an extent. This genomic redundancy, in fact, has classically been considered to be at least partially responsible for the success of polyploidy in many plant lineages (Leitch and Leitch, 2008), although the same redundancy which can buffer high-impact mutations and prevent them from being deleterious may also slow the rate of loss of deleterious alleles and fixation of beneficial alleles (Stebbins, 1971; Otto and Whitton, 2000). Homoeologous recombination events are also, of course, more common in polyploids, which provide millions of potential substrates for non-homologous recombination and formation of crossovers between two similar DNA sequences.

Homoeologous exchanges, as well as presence-absence variants and other karyotypic changes, have now been conclusively linked to phenotypic changes in many species, including polyploid crops (reviewed by Schiessl et al., 2019). In fact, a number of homoeologous exchanges (almost all examples involve duplication-deletion events, as reciprocal translocations are harder to detect) have now been demonstrated to have been selected for in crops: in *Brassica napus* (rapeseed), winter and spring crop types are differentiated by homoeologous exchanges involving major flowering time regulators such as *FLC* (Schiessl et al., 2017), and effects of homoeologous exchanges on disease resistance and glucosinolate metabolism have also been observed (Stein et al., 2017; Hurgobin et al., 2018). In allotetraploid peanut, fixed homoeologous exchanges (duplication-deletion events) were seen to generate phenotypic novelty, with direct effects on flower color (Bertioli et al., 2019). In many synthetic hybrids produced from a single homozygous individual, homoeologous exchanges lead to generation of major genetic and phenotypic novelty (Xiong et al., 2011; Spoelhof et al., 2017a; Sun et al., 2017; Li et al., 2019). Hence, homoeologous exchanges (both duplication-deletions and chromosome rearrangements) may comprise an important evolutionary substrate for divergence, speciation and adaptation in newly formed allopolyploids.

It is of interest to consider the possible relationships between HEs and the constraints on genic retention and evolution following whole genome doubling imposed by selection at the gene balance level (Birchler and Veitia, 2010, 2012, 2014). To the extent that gene content and function are equivalent among homoeologous segments, HEs would not, to a first approximation, appear to materially impact gene balance. In the case of allopolyploids, however, where there almost certainly are both functional and copy-number differences among homoeologs, it seems likely that the selective fate or survivorship of particular HEs might in part be directed by gene-balance considerations. As this is an entirely unexplored relationship, it represents a natural area for future investigation.

## Homoeologous Exchanges and Segmental Allopolyploidy

Reconciliation between the classic definition of segmental allopolyploids as “containing autopolyploid and allopolyploid segments” and modern genomic observations of genic synteny is, of course, provided by the mechanism of homoeologous exchange following allopolyploidy. A modern conception of “segmental allopolyploids” may thus include both transitional autopolyploids as well as allopolyploids that contain a mix of auto- and allopolyploid segments derived via homoeologous exchanges (duplication-deletion events; e.g., Sun et al., 2017; Leal-Bertioli et al., 2018; Bertioli et al., 2019). The dynamics of this process have now been described in numerous experimental systems.

**FIGURE 2:**
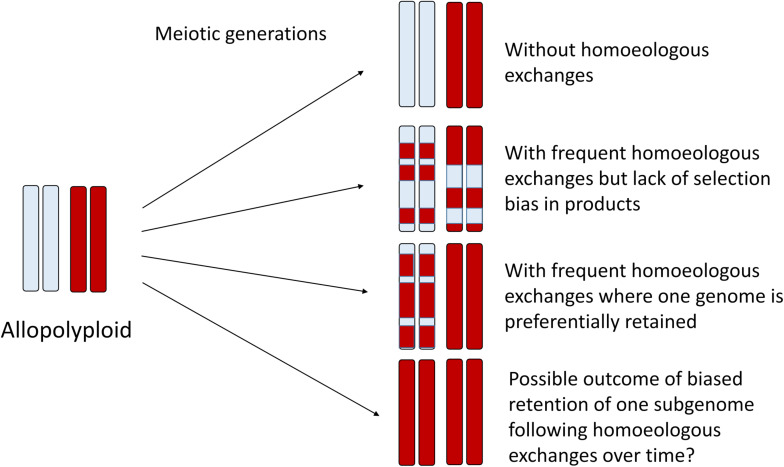
Homoeologous exchanges can generate a diverse spectrum of genomic mosaics over the generations, where some regions of the genome retain homoeologous segments and others become genomically homozygous for a single parental homoeolog, as illustrated here for one pair of homoeologous chromosomes. Thus, some regions of the genome might appear to be “autopolyploid” whereas others appear “allopolyploid.” At the population level and over time, HEs may generate highly variable progeny that may be subject to natural selection, thus fixing specific chromosomal recombinants. In the limit, directional selection may favor one progenitor homoeolog, which may thus appear to have an autopolyploid origin. Genic divergence for duplicates is expected to reflect this history.

In homoeologous exchanges, the products of a single event can be described as either “balanced” exchanges, or as “duplication-deletion” events, where the latter are hypothetically more common due to random segregation of chromatids following a homoeologous crossover event. In these “duplication-deletion” events, segments of one subgenome are deleted and replaced by segments of the other subgenome. If these events become fixed, then this genomic region is in fact “autopolyploid,” with four copies of the same subgenome, while the rest of the genome remains allopolyploid. If no selection or bias is present, this would generate a complex mosaic of genomic regions representing one or the other subgenome, or both (Figure 2), with considerable relevance to phylogenomics and phylogenetics (Edger et al., 2018). An excellent recent example of this process is provided by genomic investigations of allotetraploid peanut, in which regions of both A and B subgenomes had been replaced by copies of the other subgenome (AABB –> AAAA or BBBB) (Bertioli et al., 2019), putatively as a result of fixation of these homoeologous exchanges after a single allopolyploidization event. On the other hand, a possible outcome of biased replacement of one subgenome with the other subgenome, as has been documented to occur via fertility-based selection in some species (e.g., Gaebelein et al., 2019), could make an even more interesting pattern: an “autopolyploid” may result, but possibly one that appears to have small genomic regions introgressed from another species (Figure 2). Recently, synthetic rice polyploids formed by hybridization between *japonica* and *indica* subspecies also revealed directional loss of one subgenome through selection for the products of HEs (Zhang et al., 2019). The authors found that this “homogenization” (retention of two copies of one subgenome and loss of the corresponding homoeologous copy from the other subgenome) also altered gene expression and enhanced alternative splicing in these chromosome regions, thus suggesting a possible selective mechanism for these events. Although an interesting speculation, a uni-directional process has so far only been observed in very recent and synthetic allopolyploids. Under natural conditions, negative selective pressure against *de novo* HEs would also have to be overcome. Minority cytotype disadvantage, where individuals heterozygous for particular chromosome rearrangements have lower reproductive success, could play a role in purging novel homoeologous exchanges from populations, and likely does in most cases. Finally, it is unclear the extent to which initial conditions established at the time of allopolyploid formation, that is, the genomic features of the progenitor diploids, are determinative of the future survivorship of HEs in their derived allopolyploids. More specifically, do the same, still somewhat mysterious genomic features that are thought to be involved in the establishment of subgenome dominance (Cheng et al., 2018; Wendel et al., 2018) play an important role in the genomic distribution and selective fate of HEs? This too represents an area for future research.

## Discussion

Here we have attempted to provide a synopsis of our growing recognition that homoeologous exchange following polyploidy is a common evolutionary process leading to genomically variable progeny that can serve as substrates for natural selection. Thus, HEs comprise an important dimension of polyploid genomics, potentially representing a key mechanism of post-polyploidization diversification and speciation. This same process has implications for the inference of polyploid parentage and our understanding of the terms autopolyploidy and allopolyploidy, as well as “segmental allopolyploidy.” In this respect, phylogenetic or phylogenomic analyses will benefit from consideration of the genomic mosaicism potentially generated by HEs (Edger et al., 2018), and the possibility of conversion of a strict allopolyploid to a partially autopolyploid genome through homoeologous exchanges. The use of synthetic systems, where historical polyploidization events are “recreated” by crossing between diploid progenitor species, may help shed light on the spectrum of mechanisms and outcomes involved in the early stages of allopolyploid genome evolution (Xiong et al., 2011; Samans et al., 2017; Spoelhof et al., 2017a; Sun et al., 2017; Li et al., 2019), as may investigation of very young allopolyploids such as *Tragopogon mirus* and *T. miscellus* (Buggs et al., 2011, 2012), *Senecio cambrensis* (Ashton and Abbott, 1992; Hegarty et al., 2006), *Mimulus peregrinus* (Vallejo-Marín et al., 2015) and *Spartina anglica* (Baumel et al., 2002; Ainouche et al., 2004). In particular, better understanding of the mechanisms controlling genome stability (i.e., frequency of non-homologous recombination events and other mutations) and the possible genotypic influences on these mechanisms may prove a fruitful avenue for further investigation. Homoeologous exchanges in allopolyploids in particular may have far-reaching implications for polyploid evolution, providing evolutionary novelty, helping stabilize genomes and facilitating speciation. Our appreciation of the significance of HEs in polyploid evolution will undoubtedly be enhanced by the increasing application of genomic tools to natural (Bomblies et al., 2015; Yant and Bomblies, 2017; Marburger et al., 2019) and synthetic (Samans et al., 2017; Sun et al., 2017; Hurgobin et al., 2018; Li et al., 2019) polyploid complexes, combined with an increasing experimental focus on cytogenetic and meiotic mechanisms.

## Author Contributions

JW and AM conceptualized and wrote the manuscript. AM produced the figures with critical input from JW. Both authors contributed to the article and approved the submitted version.

## Conflict of Interest

The authors declare that the research was conducted in the absence of any commercial or financial relationships that could be construed as a potential conflict of interest.
